# CRISPR/Cas9-mediated LMP1 knockout inhibits Epstein-Barr virus infection and nasopharyngeal carcinoma cell growth

**DOI:** 10.1186/s13027-019-0246-5

**Published:** 2019-10-30

**Authors:** Haifeng Huo, Guohua Hu

**Affiliations:** Department of Otorhinolaryngology, The First Affiliated Hospital of Chongqing Medical University, Chongqing, 400016 China

**Keywords:** CRISPR/Cas9, Nasopharyngeal carcinoma, Epstein-Barr virus (EBV), LMP1, Growth

## Abstract

**Background:**

A strong association between Epstein-Barr virus (EBV) infection and nasopharyngeal carcinoma (NPC) has been widely recognized in recent decades. The aim of the present study was to investigate latent membrane protein 1 (LMP1) regulation of nasopharyngeal carcinoma (NPC) CNE-2 cell growth and then examine the effects of LMP1-knockout with CRISPR (Clustered Regularly Interspaced Short Palindromic Repeats)/Cas9on Epstein-Barr virus (EBV) infection and CNE-2 cell growth.

**Methods:**

Human NPC CNE-2 cells were infected with the recombinant LMP1- and LMP2A-carrying lentivirus, and then examined for cell growth with the colony forming assay as well as for the activation of transcription of eukaryotic translation initiation factor 4E (eIF4E) with reverse-transcriptase quantitative polymerase chain reaction (RT-qPCR) and western blot. CRISPR/Cas9-mediated knockout of LMP1 or LMP2A was performed with a single-guide RNA (sgRNA) targeting sequences within LMP1 or LMP2A. The knockout effect and the EBV proliferation were examined with RT-qPCR, western blot and cell growth assay.

**Results:**

LMP1 overexpression promoted CNE-2 cell growth, compared to LMP2A overexpression. Loss-of-function experiments confirmed that eukaryotic translation initiation factor 4E (eIF4E) upregulation mediated this effect. LMP1 knockout significantly inhibited EBV proliferation in CNE-2 cells and markedly inhibited LMP1-mediated promotion of cell growth. The knockout of either LMP1 or LMP2A blocked the eIF4E activation, which is induced either by the EBV infection or by the overexpression of LMP1 or LMP2A.

**Conclusion:**

We confirmed the LMP1-mediated promotion of NPC cell growth. Such promotion can be effectively blocked by CRISPR/Cas9-mediated LMP1 knockout. Precise LMP1 knockout might be a promising method for targeted inhibition of EBV infection and NPC cell growth.

## Background

A strong association between Epstein-Barr virus (EBV) infection and nasopharyngeal carcinoma (NPC) has been widely recognized in recent decades [1–4]. Classified as a group I carcinogen by the International Agency for Research on Cancer (IARC), EBV is detected in almost all poorly differentiated NPC cases [3, 4]. Oncogenic factors in NPC have been reduced to viral proteins such as latent membrane protein 1 (LMP1) [5, 6] and Epstein–Barr nuclear antigen 1 (EBNA1) [4, 7, 8]. The EBV-encoded small RNAs (EBERs) [9], and microRNAs are also associated with EBV-driven oncogenesis, such as miRNAs of BERTs (EBV-encoded miRNAs in PART region) [10]. Chromosomal integration of EBV genomes has been sporadically observed in NPC cells [11, 12]. Regarding the molecular mechanisms of LMP1-driven oncogenesis in NPC, multiple signaling pathways have been found involved, such as nuclear factor κB (NF-κB) [6], p38 mitogen activated protein kinase and the c-Jun N-terminal kinase (JNK) pathways [13–15]. The oncogenic role of LMP1 has been widely investigated, especially how it can promote epithelial-mesenchymal transition (EMT) and NPC cell proliferation and invasion. It positively regulates TAZ expression [16], stimulates the transcription of eukaryotic translation initiation factor 4E (eIF4E) [17], upregulates high mobility group box 1 (HMGB1), facilitates EBV-LMP1-targeted DNAzyme-induced DNA damage to cause cell cycle arrest [18], and inhibits the liver kinase B1 (LKB1)-AMP-activated protein kinase (AMPK) pathway [19].

CRISPR (Clustered Regularly Interspaced Short Palindromic Repeats)/Cas (CRISPR-associated) was recently verified as a precise and robust strategy for targeted genome editing [20–22]. Most commonly, Cas9 endonuclease and a single-guide RNA (sgRNA) are utilized to target a 20-bp-long DNA region that is complementary to the sgRNA [21, 23]. CRISPR/Cas9 technology enables loss-of-function genetic analysis of regulatory elements in the coding or non-coding region of a gene [24, 25] and robust potential for genetic modification. The CRISPR/Cas9 system has been applied to develop various antiviral strategies [26, 27], including against human herpesvirus (HHV) [28, 29]. This strategy is more effective than other antivirus methods, particularly for viruses that integrate into human chromosomes, such as human immunodeficiency virus (HIV) and human papillomavirus (HPV) [30]. It was also demonstrated to effectively eliminate EBV genomic episomes from latent cells [31, 32].

The present study aimed to evaluate the effect of CRISPR/Cas9-mediated knockout of LMP1 or LMP2A on CNE-2 cell proliferation.

## Methods

### Cell culture and CRISPRS-Cas9 treatment

Human NPC CNE-2 cells were cultured in RPMI-1640 medium (Thermo Fisher Scientific, Waltham, MA, USA), with 10% fetal bovine serum (FBS, Thermo Fisher Scientific, Waltham, MA, USA) and with 1x penicillin/streptomycin solution (Invitrogen/Thermo Fisher Scientific). Cell incubation was performed at 37 °C under 5% carbon dioxide (CO_2_). To overexpress LMP1 or LMP2A, 85%-confluent CNE-2 cells were infected with the recombinant LMP1- or (and) LMP2A-carrying lentivirus (Cqwestern. Chongqing, PR China), and with chloramphenicol acetyl transferase (CAT)-carrying lentivirus, cloned from pcDNA3.1/CAT, as the up control. CNE-2 cells were infected with a multiplicity of infection (MOI) of 10 and then were selected under puromycin pressure (2 μg/ml) for ten days. CNE-2 cells were infected with EBV (0.01 MOI), before the examination of the CRISPR/Cas9-mediated inhibition to virus proliferation, to LMP1/ LMP2A expression, or to eIF4E activation,

For CRISPR/Cas9-mediated knockout of LMP1 or LMP2A, the sgRNA targeting sequences were designed targeting potential 20-base-long sites on the EBV genome with the online gRNA design tool (Crispr.mit.edu). sgRNA sequences were listed as follows: LMP1 (targeting sequence 5′- TTG AGA GGG GCC CAC CGG GCC CG-3′ [14–36 in LMP1 coding region] and 5′-CGC CTT TGA TGA CAG ACG GAG GC-3′ [1007–1029 in LMP1 coding region]); LMP2A (targeting sequence 5′-GCC GTT ACG TGT CCC GGG TGG TC-3′ [14–36 in LMP2A coding region] and 5′-TGC CTC AGT GGA CTT CTC ACC GC-3′ [1231–1253 in LMP2A coding region]). The sgRNA sequences were inserted into the sgRNA-expression vector PX459, and the gRNA-PX459 recombinant plasmids were co-transfected with gRNA-Cas9 co-expression plasmid PX459 using Lipofectamine 3000 (Invitrogen/Thermo Fisher Scientific). Positive cell clones were selected from the transfected cells at 48 h post transfection under the pressure of 1 μg/ml puromycin for two weeks, via serial passages.

### Reverse-transcriptase quantitative polymerase chain reaction (RT-qPCR)

Total mRNA was extracted from CNE-2 cells with a magnetic mRNA Isolation Kit (New England Biolabs, Ipswich, MA USA) according to the manufacturer’s protocol. Equivalent amount of mRNA sample was transcribed into cDNA with a GoScript™ Reverse Transcriptase (Promega, Madison, WI, USA). EBV viral DNA was purified with genomic DNA isolation kits (Qiagen, Venlo, Netherlands) under the guidance of the kit’s instruction. The cDNA was examined by RT-qPCR (PrimeScript™ RT Reagent Kit, Takara, Tokyo, Japan) to determine the relative mRNA levels of LMP1, LMP2A, and eIF4E using β-actin for gene expression normalization with LightCycler 2.0 (Roche Diagnostics, Risch-Rotkreuz, Switzerland). To quantify EBV genomic DNA in CNE cells after EBV infection, the levels of EBNA1 and OriP were quantified with RT-qPCR. The EBNA1 or OriP DNA level is presented as a relative value to each DNA level at 0-h post-EBV infection.

### Western blot analysis

CNE-2 cells were lysed with Cell Lysis Buffer (Cell Signaling Technology, Danvers, MA, USA) and supplemented with protease inhibitor cocktail kit (Roche Biochemicals, Penzberg, Germany). Protein samples were separated by 10% sodium dodecyl sulfate-polyacrylamide gel electrophoresis and then transferred onto polyvinylidene fluoride membranes. The membrane was incubated with primary antibodies for LMP1- (1:2000, Abcam, Cambridge, UK), LMP2A- (1: 1000, Abcam), eIF4E-specific (1: 1000, Abcam) or β-actin (1: 3000, Sino Biological, Wayne, PA, USA). Horseradish peroxidase (HRP)-linked secondary antibodies (Jackson ImmunoResearch, West Grove, PA, USA) and enhanced chemiluminescence (Thermo Fisher Scientific) were utilized to visualize the specific binding of the HRP-linked second antibody to first antibody-protein complexe, under the UVP BioSpectrum 500 imaging system (UVP, Upland, CA, USA).

### Colony-forming assay and cell growth assay

CNE-2 cells, with LMP1- or LMP2A-overexpression or with LMP1- and/or LMP2A-knock out, were seeded and incubated in six-well plates for three days. Colony formation was observed under an Olympus BX60 microscope (Olympus, Tokyo, Japan). Cell growth was assessed with CCK-8 assays (DOJINDO, Tokyo, Japan). Briefly, CNE-2 cells were suspended (100 μl/well) in a 96-well plate and incubated in a humidified incubator (at 37 °C with 5% CO_2_) for 24, 48, or 72 h. Next, 10 μl CCK-8 solution was added to each well for 2-h’s incubation at 37 °C, and then absorbance at 450 nm was measured using a microplate reader (Bio-Rad, Hercules, CA, USA).

All experiments were approved by the Ethics Committee of Chongqing Medical University (20170121).

### Statistical analysis

Quantitative results are presented as mean ± standard deviation and were analyzed with two-tailed Student’s t-tests. Differences were considered significant at *p* < 0.05.

## Results

### EBV LMP1 promotes NPC CNE-2 cell growth in an eIF4E-dependent fashion

**Fig. 1:**
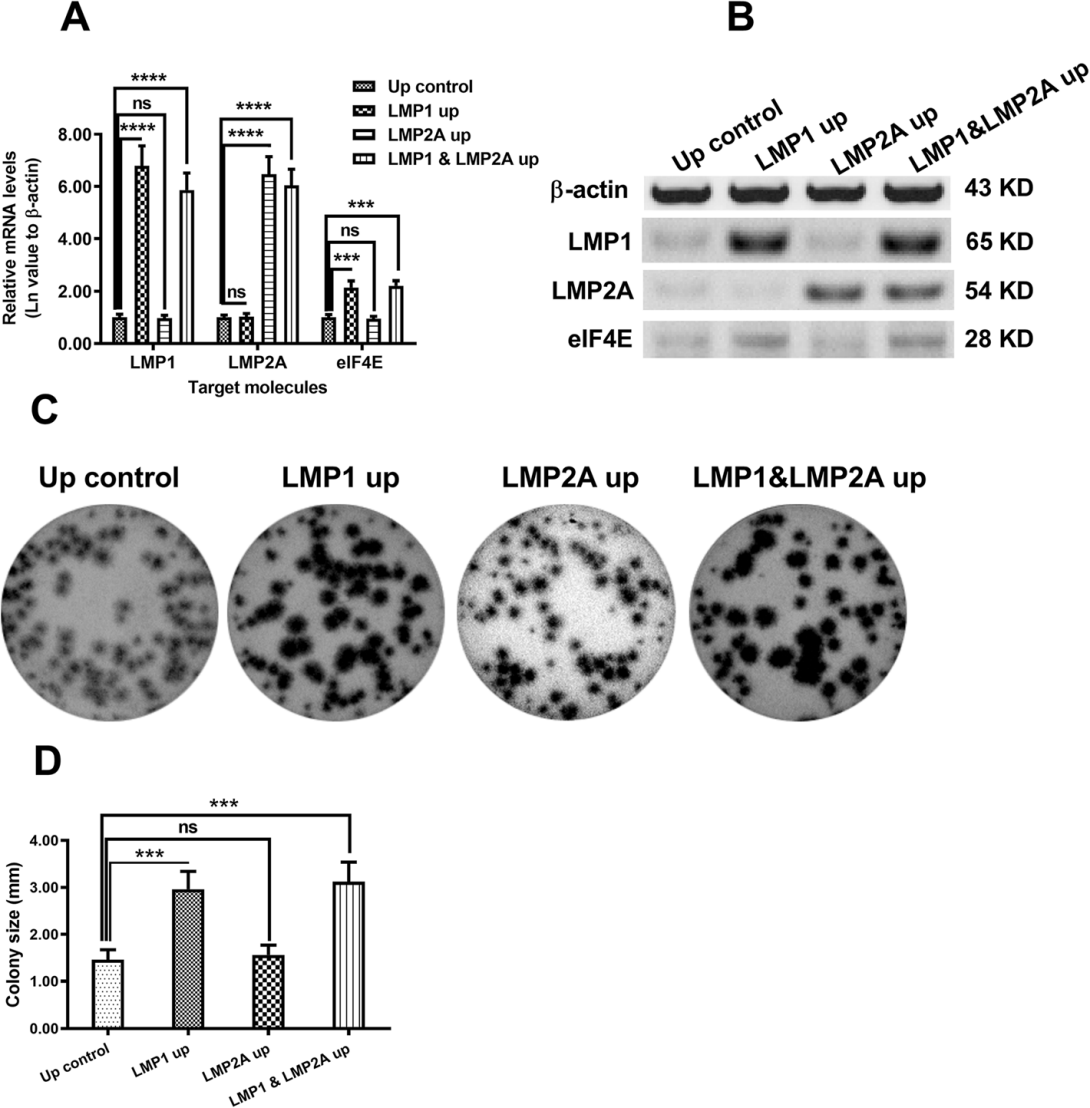
LMP1 overexpression regulates CNE-2 cell growth. **a** and **b**: Real-time quantitative PCR analysis (**a**) and western blotting (**b**) of mRNA and protein levels of LMP1, LMP2A, and eIF4E in the control, LMP1-overexpressing, LMP2A-overexpressing or both LMPs-overexpressing CNE-2 cells; **c** and **d**: Colony formation and colony size for CNE-2 cells after 3 days of incubation, measured via colony forming assays. Up control, control; LMP1 up, LMP1-overexpressed; LMP2A up, LMP2A-overexpressed; LMP1&LMP2A up, both LMP1- and LMP2A overexpressed. Each result was repeated independently in triplicate. ***p < 0.001; ****p < 0.0001; ns, no significance

We overexpressed LMP1 or LMP2A in CNE-2 cells with lentiviral vectors, using CAT-carrying lentivirus as the up-regulation control (abbreviated as up control). Figure 1a shows dramatically increased mRNA levels of LMP1 and LMP2A in the LMP1 up-regulation (LMP1 up) and LMP2A up-regulation (LMP2A up) groups, respectively, in comparison with the Up-control group (both *p* < 0.001). Both LMP proteins were upregulated in CNE-2 cells following LMP1- and LMP2A-lentivirus infection (both *p* < 0.0001, Fig. 1b). Also, higher eIF4E mRNA levels were observed in LMP1-overexpressing CNE-2 cells with or without LMP2A overexpression (*p* < 0.001 Fig. 1a). Higher eIF4E protein level was also found in CNE-2 cells (Fig. 1b). To evaluate the LMP regulation of NPC cell growth, we performed colony-forming assays in CNE-2 cells followingLMP1- and LMP2A-lentivirus infection. As indicated in Fig. 1c, significantly larger colonies were formed by CNE-2 cells after LMP1-lentivirus infection compared to LMP2A- as well as LMP1&LMP2A-lenticirus infection (*p* < 0.001 for the LMP1 up and LMP1&LMP2A up groups, Fig. 1d).

**Fig. 2:**
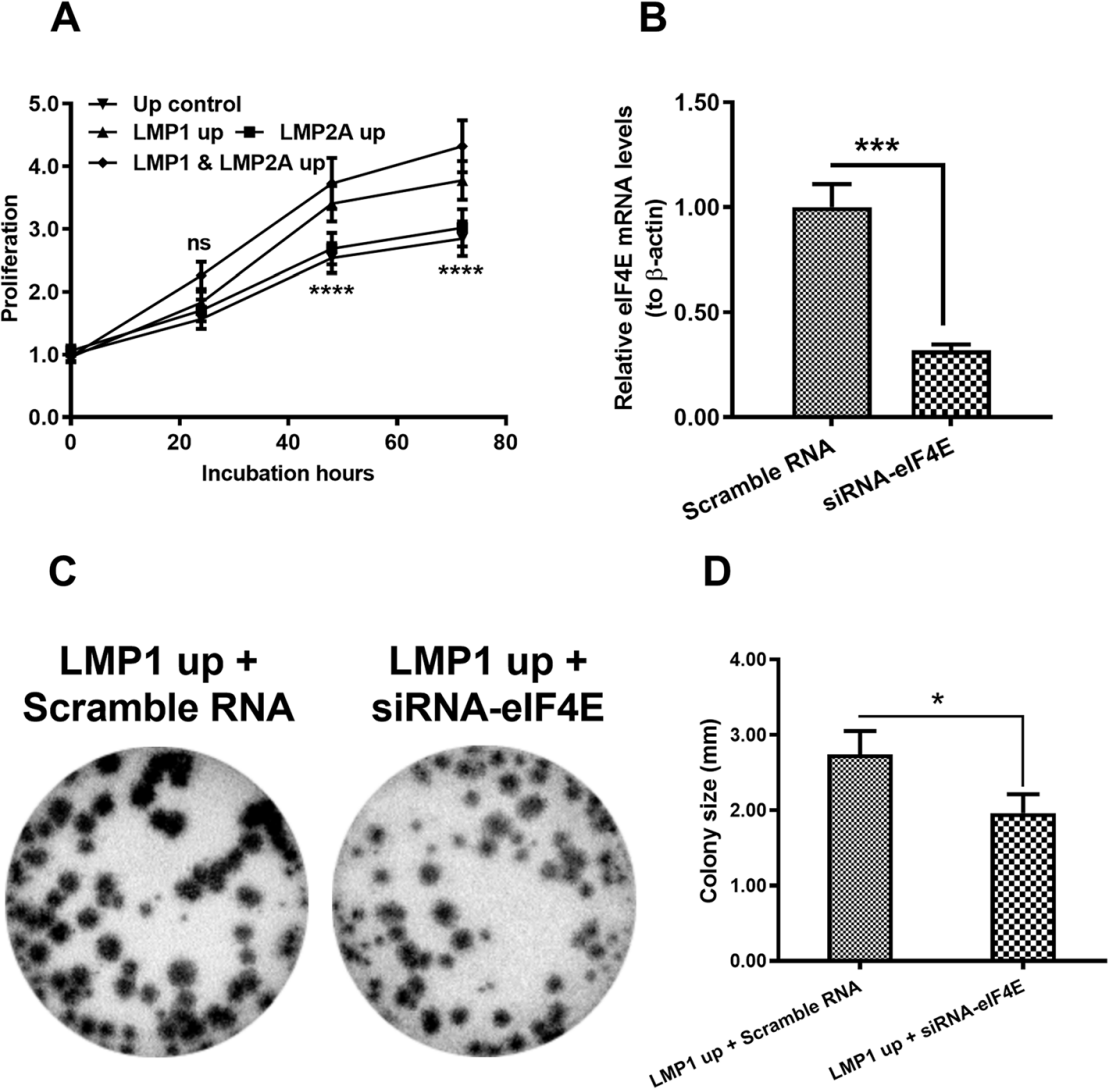
Dependence of eukaryotic translation initiation factor 4E on the LMP1-mediated regulation on CNE-2 cell growth. **a**: Growth curves of the four groups of CNE-2 cells incubated for 24, 48, and 72 h. **b**: Relative eIF4E mRNA levels in LMP1-overexpressing CNE-2 cells after the eIF4E knockdown. **c** and **d**: Colony formation and colony size for LMP1-overexpressing CNE-2 cells after the eIF4E knockdown. Scramble RNA, negative control for siRNA-eIF4E; siRNA-eIF4E: eIF4E-specific siRNA. Each result was repeated independently in triplicate. *p < 0.05; ***p < 0.001; ****p < 0.0001; ns, no significance

Besides, growth curves confirmed that LMP1 increases cell growth (*p* < 0.0001, Fig. 2a). We evaluated whether LMP1-mediated promotion of CNE-2 cell growth is dependent on eIF4E. Effective knockdown of eIF4E (*p* < 0.001, Fig. 2b) significantly inhibited growth promotion in CNE-2 cells (*p* < 0.05, Fig. 2 c and d). Thus, LMP1-mediated promotion of NPC cell growth was observed in CNE-2 cells and was at least partly dependent on eIF4E.

### CRISPR/Cas9-mediated knockout of LMP1 or LMP2A inhibits EBV replication

**Fig. 3:**
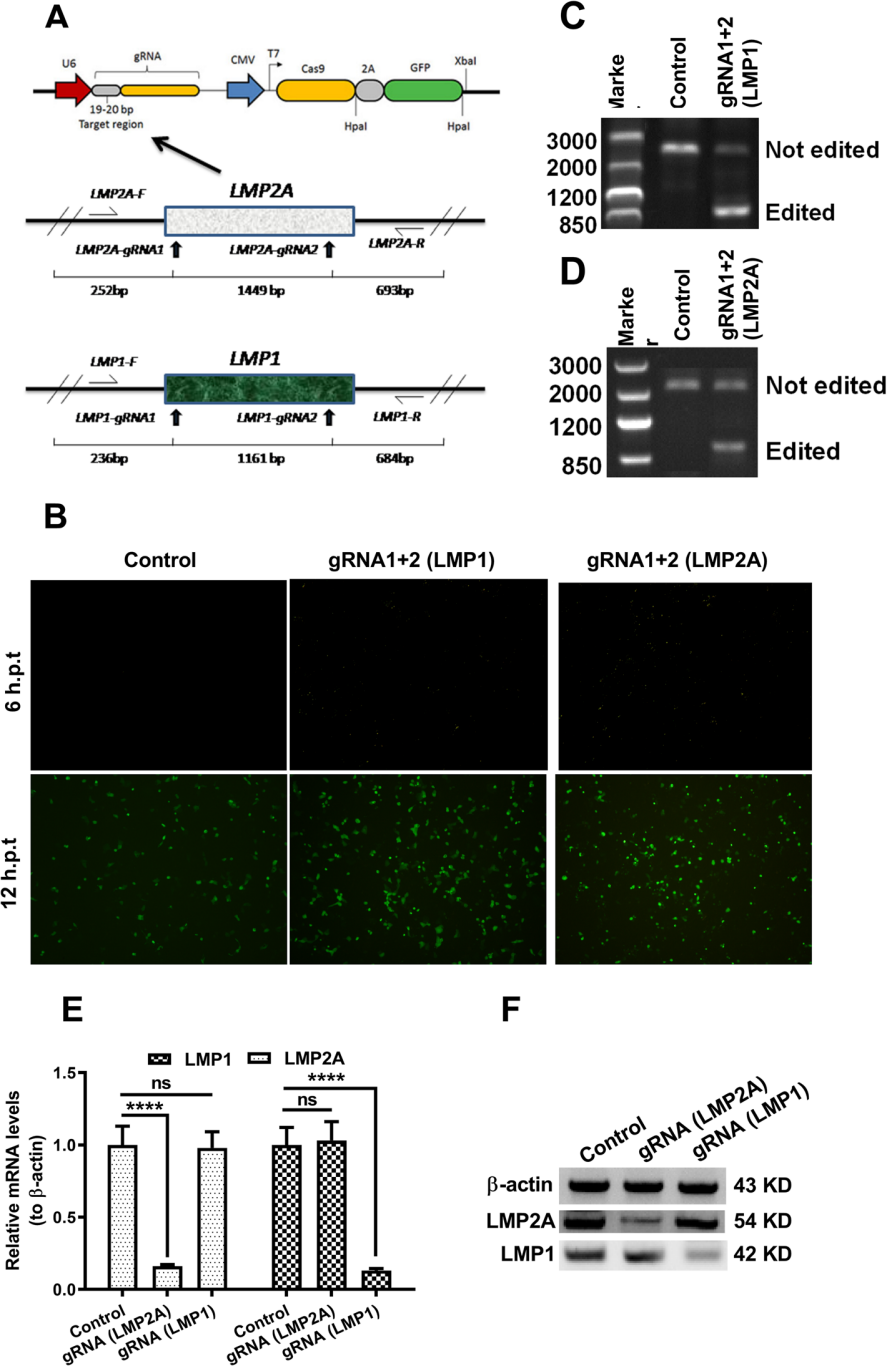
Targeted CRISPR/Cas9 inhibition of EBV LMP1 and LMP2A genes in CNE-2 cells. **a**: Schematic depicting the target sites CRISPR/Cas9 editing in LMP2A (upper) and latent membrane protein 1 (LMP1) (lower) genes of the EBV genome. The target sites of gRNAs and binding sites of specific primers (LMP1-F, LMP1-R, LMP2A-F, and LMP2A-R) are indicated with arrows. **b**: Green fluorescence intensity in CNE-2 cells transfected with the LMP1-CRISPR/Cas9 or LMP2A-CRISPR/Cas9 plasmid for 6 and 24 h. **c** and **d**: Editing effect of CRISPR/Cas9. CNE-2 cells were transfected with gRNA-Cas9 co-expression plasmids for 48 h, and the total genomic DNA was extracted for PCR analysis withLMP1-(up) and LMP2A-specific (down) primers. **e** and **f**: Real-time quantitative PCR analysis (**e**) and western blotting (**f**) of LMP1 and LMP2A in ‘LMP1 up’ CNE-2 cells that were transfected with LMP1- or LMP2A-specific gRNA-Cas9 co-expression plasmids for 48 (for mRNA quantification) or 72 (for western blotting) hours. Experiments were independently performed in triplicate. *p < 0.05; ***p < 0.001; **** *p* < 0.0001; ns, no significance

To explore the potential of CRISPR/Cas9-mediated knockout of EBV oncogenic proteins, LMP1- and LMP2A-targeted gRNA sites and the schematic diagram of the constructed LMP1- and LMP2A-editing systems were shown in Fig. 3a. The conservation of the target sites was checked via aligning the target sequences with other viral coding sequences. The successful insertion and effective transfection of either gRNA1 + 2 (LMP1) / gRNA1 + 2 (LMP2A) or the control plasmid were indicated by the expression of green fluorescence protein, at the 12-h post transfection (Fig. 3b). The editing effectiveness of both LMP targets is shown in Fig. 3 c and d under the same transfection efficiency.

**Fig. 4:**
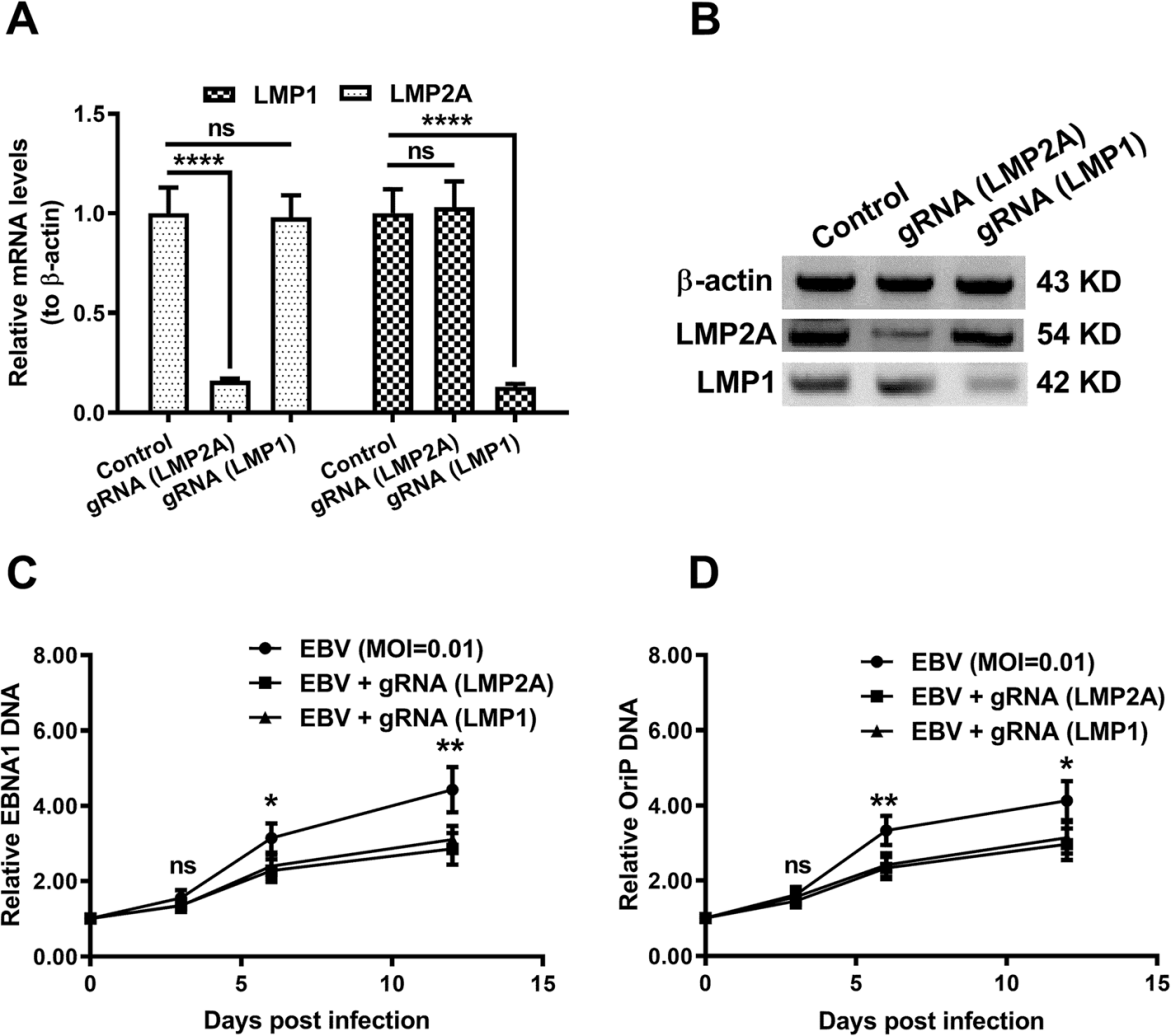
Regulation by the CRISPR/Cas9-mediated knockout of LMP1 or LMP2A on the proliferation of EBV in CNE-2 cells. **a**: Real-time quantitative PCR analysis (**a**) and western blotting (**b**) of LMP1 and LMP2A in the ‘LMP1 up’ CNE-2 cells, which were transfected with LMP1- or LMP2A-specific gRNA-Cas9 co-expression plasmids for 48 (for mRNA quantification) or 72 (for western blotting) hours; **c** and **d**: Relative EBV growth in the ‘LMP1 up’ CNE-2 cells, post the CRISPR/Cas9-mediated knockout of LMP1 or LMP2A, with a multiplicity of infection (MOI) of 0.01 for 0, 3, 6, or 12 days; virus growth was determined via the quantification of viral DNA levels of EBNA1 (**c**) and OriP (**d**). Each value was an average for triple independent results. Statistical significance was showed as * p < 0.05, ** *p* < 0.01, **** *p* < 0.0001, ns: no significance

Both mRNA (*p* < 0.0001 respectively, Fig. 4a) and protein (Fig. 4b) levels of LMP1 and LMP2A were markedly knocked down in CNE-2 cells at 24 h after EBV infection. In addition, the regulation by LMP1 or LMP2A editing on virus replication was also examined. The virus growth was evaluated with the viral DNA levels of EBNA1 and OriP. A significant reduction of EBNA1 (Fig. 4c) and of OriP (Fig. 4d) DNA (*p* < 0.05 or *p* < 0.01 for 6 or 12-days post-infection) was caused by the knockout of either LMP. These results confirmed that the LMP1 or LMP2A knockout inhibited the EBV replication.

### CRISPR/Cas9mediated knockout of LMP1 inhibits CNE2 cell growth and eIF4E activation

**Fig. 5:**
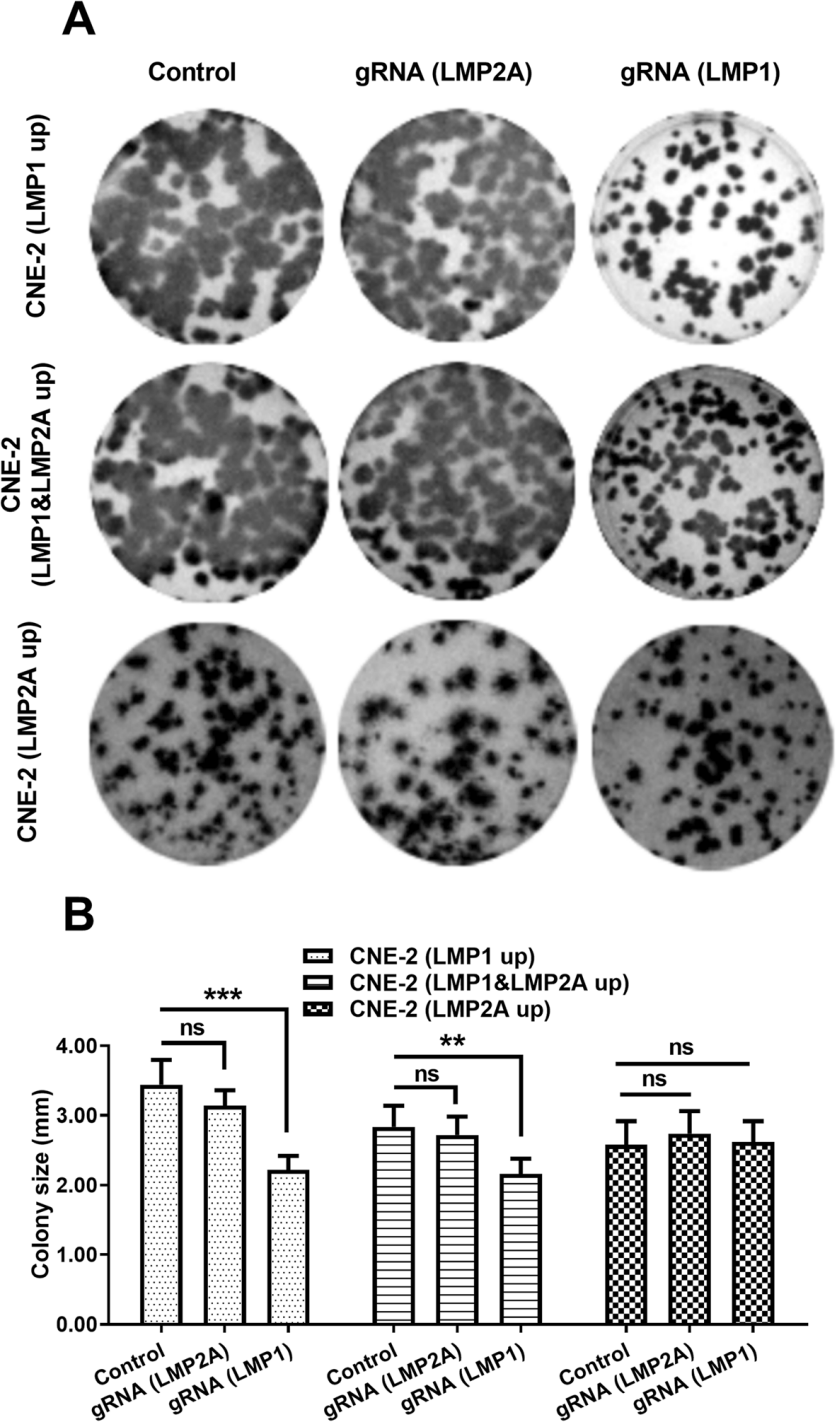
CRISPR/Cas9-mediated knockout of LMP1 or LMP2A regulates CNE-2 cell growth. **a** and **b**: Colony formation (**a**) and size (**b**) of CNE-2 cells following the targeted knockout of LMP1 or LMP2A, via transfection with the LMP1- or LMP2A-specific gRNA-Cas9 co-expression plasmid. Each experiment was independently replicated three times. ** *p* < 0.01; *** *p* < 0.001; ns, no significance

**Fig. 6:**
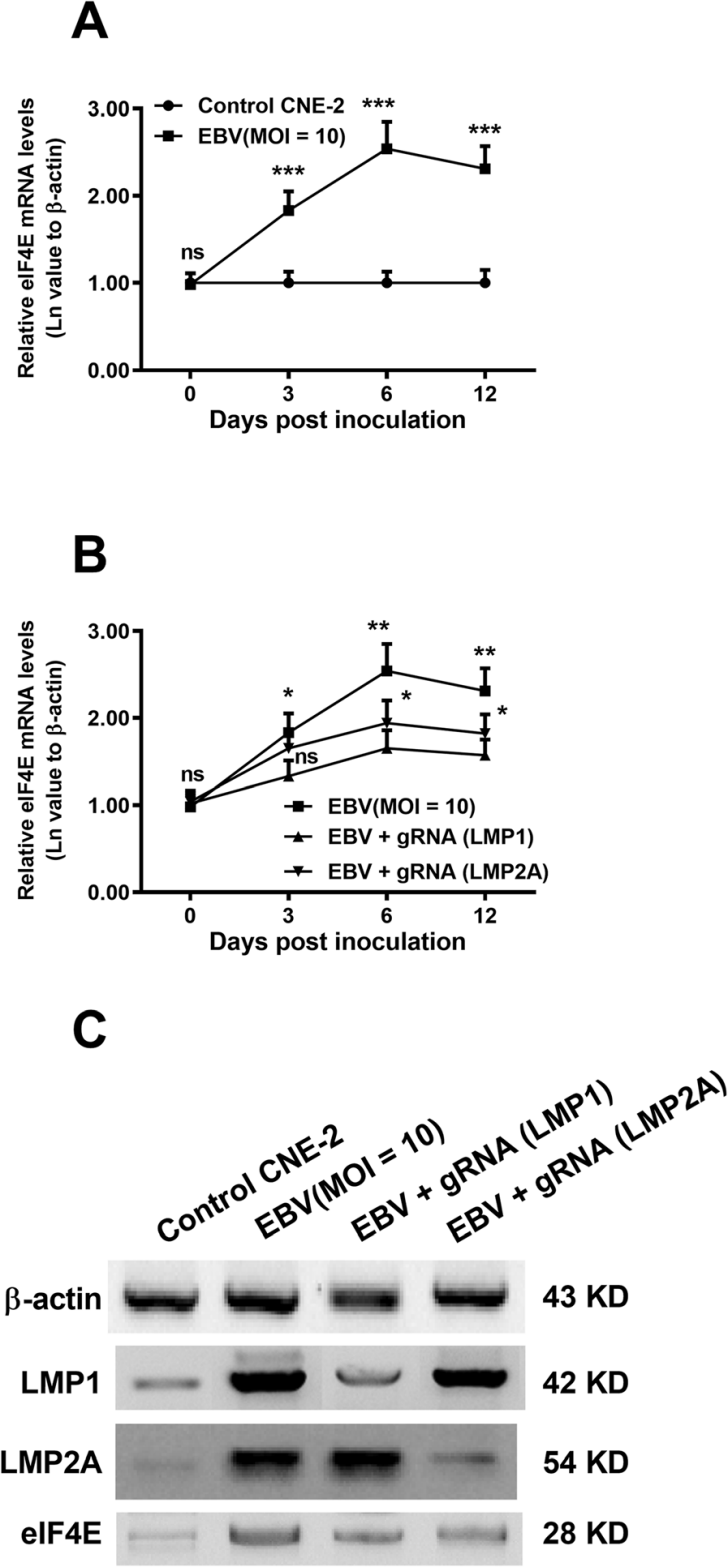
CRISPR/Cas9-mediated knockout of LMP1 or LMP2A down-regulates the eIF4E activation in the EBV-infected CNE-2 cells. CRISPR/Cas9-mediated knockout of LMP1 or of LMP2A was performed in the CNE-2 cells, post the infection with 10 MOI EBV, then the mRNA and the protein levels of LMP1, LMP2A, and eIF4E were examined by the method of Real-time quantitative PCR analysis (**a**) or of western blotting (**b**). Experiments were independently repeated in triplicate. ** *p* < 0.01; *** *p* < 0.001; ns, no significance

CRISPR/Cas9-mediated knockout of both LMP1 and LMP2A was performed before measuring CNE-2 cell growth with colony forming assays. LMP1 knockout markedly decreased colony size in LMP1 up cells (*p* < 0.001, the right column, Fig. 5 a, b). However, LMP2A knockout did not have any effect (the middle column, Fig. 5 a, b). Significant colony size reduction was also observed in LMP1&LMP2A up cells after LMP1 knockout (*p* < 0.01) rather than LMP2A knockout. Furthermore, knockout of either LMP did not influence colony size in LMP2A up cells. Therefore, CRISPR/Cas9-mediated knockout of LMP1 inhibited CNE-2 cell growth. In addition, we also examined the regulation by the CRISPR/Cas9-mediated knockout of LMP1 or of LMP2A on the eIF4E activation in CNE-2 cells. As indicated in Fig. 6a, the eIF4E mRNA was upregulated by the EBV infection from 3 to 12 days post-infection (p < 0.001). However, the eIF4E mRNA upregulation was blocked by either LMP1 knockout (*p* < 0.05 or p < 0.01) or by LMP2A knockout (p < 0.05, Fig. 6b). Such regulation was also confirmed at protein level. Not only LMP1 and LMP2A but also eIF4E were downregulated by the knockout of either LMP1 or LMP2A during EBV infection (Fig. 6c).

## Discussion

LMP1 upregulation was demonstrated to confer a proliferative advantage and the ability to resist apoptosis to NPC cells, via the interaction of LMP1 with cell cycle-related molecules such as NF-κB, JNK, and phosphatidylinositide 3-kinases (PI3K) [33]. In the present study, we confirmed in vitro that LMP1 promoted the proliferation of NPC CNE-2 cells at least partly via eIF4E upregulation. Previous studies indicated that LMP1 stimulated eIF4E transcription [17] in LMP1-harboring NPC B95–8 cells. However, other signaling pathways, including HMGB1 [34], DNAzyme [18] and LKB1-AMPK [19] might also be involved. EBV infection was shown to cause a statistically significant overexpression of HMGB1 in NPC tissues, in association, in association with the malignant status of NPC. The HMGB1 upregulation was demonstrated to induce NPC cells proliferation, RAGE-dependently [34]. DNAzyme (DZ) 1 [18] and LKB1-AMPK pathway [19] have also been found involved in such process via regulating cell cycle [18] or via regulating the proliferation and transformation of epithelial cells [19].

Advanced NPC has a high mortality rate [35]. Due to the anatomical complexity of the nasopharynx, surgical resection for NPC is technically challenging. Despite sensitivity to radiotherapy at early stages and encouraging clinical outcomes, it is still a challenge to administer for the same reason [36]. The roles of induction and adjuvant chemotherapy remain to be well defined [37, 38]. Given the overwhelming evidence of a robust oncogenic effect of EBV infection, targeting EBV infection might be a potential therapy. The present study and previous reports [39, 40] confirm the effective inhibition that anti-EBV infection strategies have on NPC cell growth. Notably, the coding sequence of the two latent viral proteins, LMP1 and LMP2A, integrates into the host genome and pose the ontogenetic effect for a lifelong time. It is a vital strategy to eliminate the two oncogenic genes from contaminated cells.

The CRISPR/Cas9 system derives from a prokaryotic antiviral immune system and can also effectively eliminate HPV genomic episomes from latent cells [28, 29]. Prevention of EBV infection by CRISPR/Cas9 mutagenesis was demonstrated in EBV-transformed B-lymphoblastoid cells [41, 42], and this was directly related to LMP1 [43]. We constructed here LMP1- and LMP2A-targeted CRISPR/Cas9 systems to knockout the expression of either viral protein in CNE-2 cells, and we confirmed the anti-cancer effect of CRISPR/Cas9-mediated LMP1 knockout in CNE-2 cells. Such anti-cancer effect was associated with the blockage of eIF4E activation,

Our promising results indicate that CRISPR/Cas9-mediated knockout of either LMP1 or LMP2A can prevent EBV infection of CNE-2 cells. LMP1-mediated promotion to NPC cell growth can be effectively blocked by CRISPR/Cas9-mediated LMP1 knockout. Precise LMP1 knockout might be a promising method for targeted inhibition of EBV infection and NPC cell growth.

## Data Availability

All data in this manuscript were available upon a request.

## Abbreviations

CAT: Chloramphenicol acetyl transferase
c-JNK: c-JunN-terminal kinase
CRISPR: Clustered Regularly Interspaced Short Palindromic Repeats
EBERs: EBV-encoded small RNAs
EBNA1: Epstein–Barr nuclear antigen 1
EBV: Epstein-Barr virus
eIF4E: Eukaryotic translation initiation factor 4E
EMT: Epithelial-mesenchymal transition
FBS: Fetal bovine serum
HIV: Human immunodeficiency virus
HMGB1: High mobility group box 1
HPV: Human papillomavirus
LKB1: Liver kinase B1
LMP1: Latent membrane protein 1
NF-κB: Nuclear factor kappa-light-chain-enhancer of activated B cells
NPC: Nasopharyngeal carcinoma
RT-qPCR: Real-time quantitative polymerase chain reaction
sgRNA: single-guide RNA
STAT: Signal Transducer and Activator of Transcription
